# Endocrine Protection of Ischemic Myocardium by FGF21 from the Liver and Adipose Tissue

**DOI:** 10.1038/srep02767

**Published:** 2013-09-26

**Authors:** Shu Q. Liu, Derek Roberts, Alexei Kharitonenkov, Brian Zhang, Samuel M. Hanson, Yan Chun Li, Li-Qun Zhang, Yu H. Wu

**Affiliations:** 1 Biomedical Engineering Department, Northwestern University, Evanston, IL 60208, USA; 2 Diabetes Research, Lilly Research Laboratories, Indianapolis, IN 46285, USA; 3 Department of Medicine, Division of Biological Sciences, The University of Chicago, Chicago, IL 60637, USA; 4 Rehabilitation Institute of Chicago, Chicago, IL 60611, USA

## Abstract

Myocardial ischemia, while causing cardiomyocyte injury, can activate innate protective processes, enhancing myocardial tolerance to ischemia. Such processes are present in not only the heart, but also remote organs. In this investigation, we demonstrated a cardioprotective process involving FGF21 from the liver and adipose tissue. In response to myocardial ischemia/reperfusion injury in the mouse, FGF21 was upregulated and released from the hepatic cells and adipocytes into the circulation and interacted with FGFR1 in cardiomyocytes under the mediation of the cell membrane protein β-Klotho, inducing FGFR1 phosphorylation. This action caused phosphorylation of the signaling molecules PI3K p85, Akt1, and BAD, thereby reducing caspase 3 activity, cell death, and myocardial infarction in association with improvement of myocardial function. These observations suggest that FGF21 is upregulated and released from the liver and adipose tissue in myocardial injury, contributing to myocardial protection by the mediation of the FGFR1/β-Klotho–PI3K–Akt1–BAD signaling network.

Myocardial ischemia, while causing injury of cardiomyocytes and impairment of cardiac function, can activate innate protective processes, enhancing cardiomyocyte tolerance to ischemia and promoting myocardial repair. Such protective processes have been found within the cardiac system. Injured and activated cells in the ischemic myocardium may express and/or release secretory factors including adenosine^1^,^2^, opioids^3^,^4^, bradykinin^5^,^6^, and VEGF^7^,^8^,^9^,^10^ to protect the ischemic myocardium from injury, and cardiac resident stem cells may be activated to promote myocardial regeneration^11^,^12^,^13^,^14^. Myocardial ischemia may also cause global cardioprotective responses. One example is myocardial ischemia-induced mobilization of bone marrow cells that promote myocardial repair and regeneration^15^,^16^,^17^,^18^,^19^. Several recent investigations have demonstrated that the liver can respond to ischemic myocardial injury to upregulate and release cardioprotective secretory proteins^20^,^21^,^22^,^23^,^24^, including fibroblast growth factor 21 (FGF21), as identified by microarray-based gene expression profiling^21^ and protein analyses^24^. FGF21 was able to act on ischemic cardiomyocytes to mitigate acute myocardial injury^24^. The present investigation was designed to test a signaling mechanism that mediates FGF21-based myocardial protection in the mouse model of myocardial ischemia/reperfusion injury.

FGF21 is one of the 22 FGF family proteins^25^,^26^,^27^ and is found in the liver and, to a lesser level, in the adipose tissue^27^,^28^ and pancreatic β islet cells^29^. Whereas the majority of the FGF family proteins regulate cell proliferation and differentiation in embryogenic and pathogenic processes, FGF21 has been reported to stimulate cell glucose intake^30^,^31^, induce insulin expression in the pancreatic β cells^32^, and regulate lipid metabolism^31^,^33^,^34^,^35^. FGF21 interacts potentially with all known protein tyrosine kinase-coupled FGF receptors (FGFRs), including FGFR1, FGFR2, FGFR3, and FGFR4, but preferentially binds to FGFR1^32^,^36^,^37^,^38^, an action requiring the mediation of the cell membrane protein β-Klotho^39^,^40^,^41^,^42^. This action was supported by recent studies addressing FGF21-mediated regulation of lipid metabolism in adipocytes^38^,^39^. A protein tyrosine kinase-coupled receptor is known to activate potentially the class IA members of the phosphatidylinositide 3-kinase (PI3K) family and Akt1, molecules supporting cell survival in injury^8^,^43^,^44^,^45^,^46^,^47^. One of the potential processes downstream to these molecules is Akt1-induced phosphorylation of the apoptosis regulatory factor Bcl-XL/Bcl-2-associated death promoter (BAD), an action suppressing cell apoptosis^8^,^29^,^48^. The involvement of BAD in this signaling event is further supported by the observation that dephosphorylation of BAD by the calcium-dependent serine-threonine phosphatase calcineurin triggers mitochondrion-mediated cell apoptosis^49^. As apoptosis is a process involved in myocardial ischemia/reperfusion injury^50^,^51^, apoptosis suppression is an effective approach for myocardial protection. In this investigation, we intended to test the hypothesis that FGF21 protects cardiomyocytes from injury via the mediation of the FGFRs/β-Klotho–PI3K–Akt1–BAD signaling network in myocardial ischemia/reperfusion injury.

## Results

### FGF21 upregulation in the liver and adipose tissue

**Figure 1:**
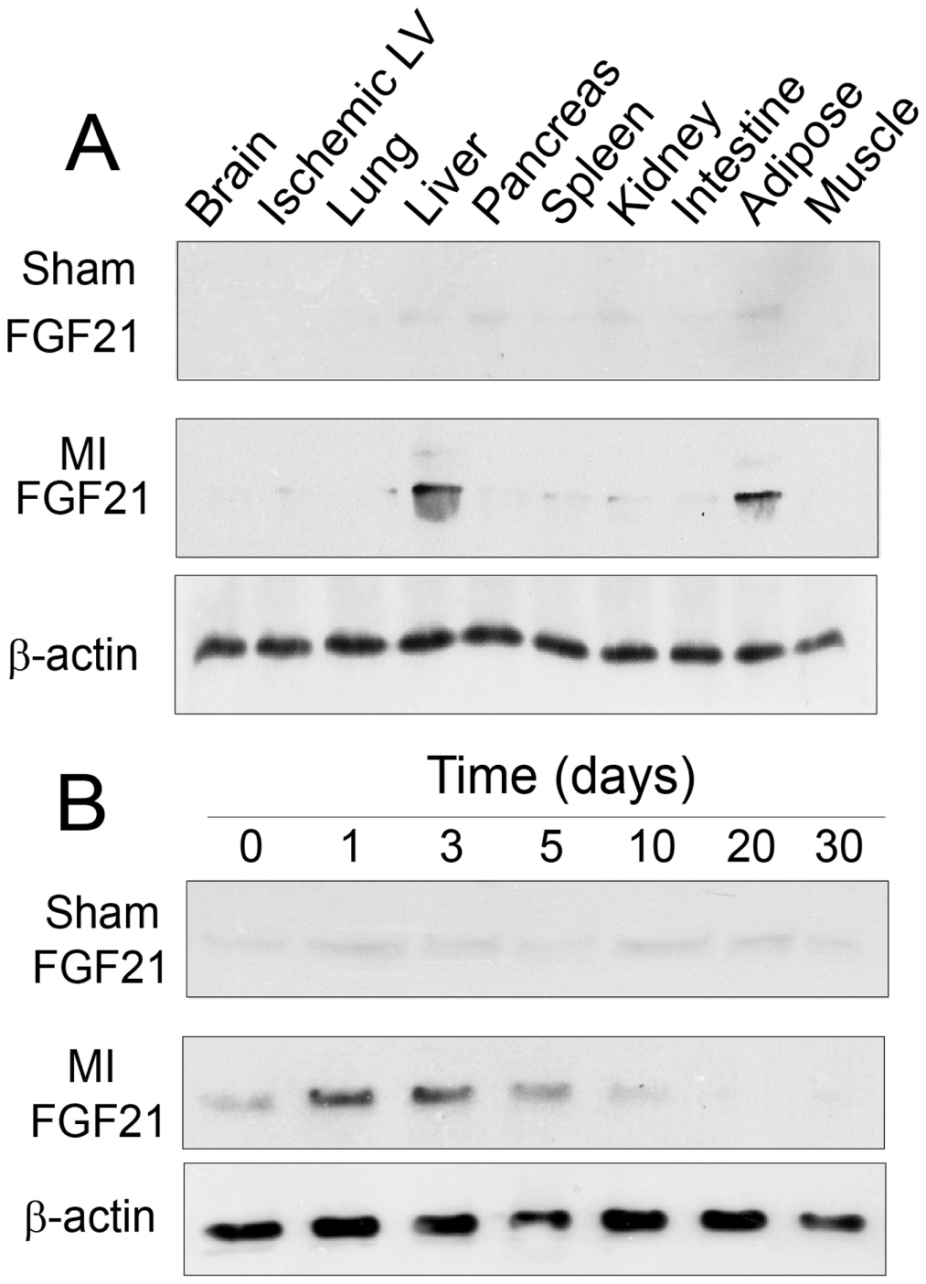
FGF21 expression in the liver and adipose tissue in response to myocardial ischemia/reperfusion injury. (A) Immunoblot analyses of FGF21 expression in selected organs and tissues at 24 hrs following sham operation or myocardial ischemia/reperfusion injury. (B) Time course of FGF21 expression in adipocytes from mice with sham operation or myocardial ischemia/reperfusion injury by immunoblot analyses. Note that cropped gel images are used in this figure and the gels were run under the same experimental conditions.

FGF21 is a secretory protein upregulated in the liver in response to myocardial ischemia^21^,^24^. It is possible that myocardial ischemia induces FGF21 expression in other organs and tissues. To test this possibility, we assessed the relative expression of FGF21 in the brain, ischemic myocardium, lung, liver, pancreas, spleen, kidney, small intestine, adipose tissue, and skeletal muscle of wild-type mice with sham-operation and myocardial ischemia/reperfusion injury. As shown in Fig. 1A, FGF21 was expressed at a low level in the selected organs of sham control mice. Myocardial injury caused FGF21 upregulation in the liver and adipose tissue, but not in other organs. Primary cell types expressing FGF21 were hepatocytes in the liver^24^ and adipocytes in the adipose tissue (Fig. 1B). The time course of FGF21 expression was similar between the two cell types (see ref. 24 and Fig. 1B). Changes in the serum level of FGF21 in myocardial ischemia were presented in a previous report^24^. These observations suggested a possible endocrine cardioprotective mechanism involving FGF21 from the liver and adipose tissue.

### Expression of FGFRs and β-Klotho in cardiomyocytes

**Figure 2:**
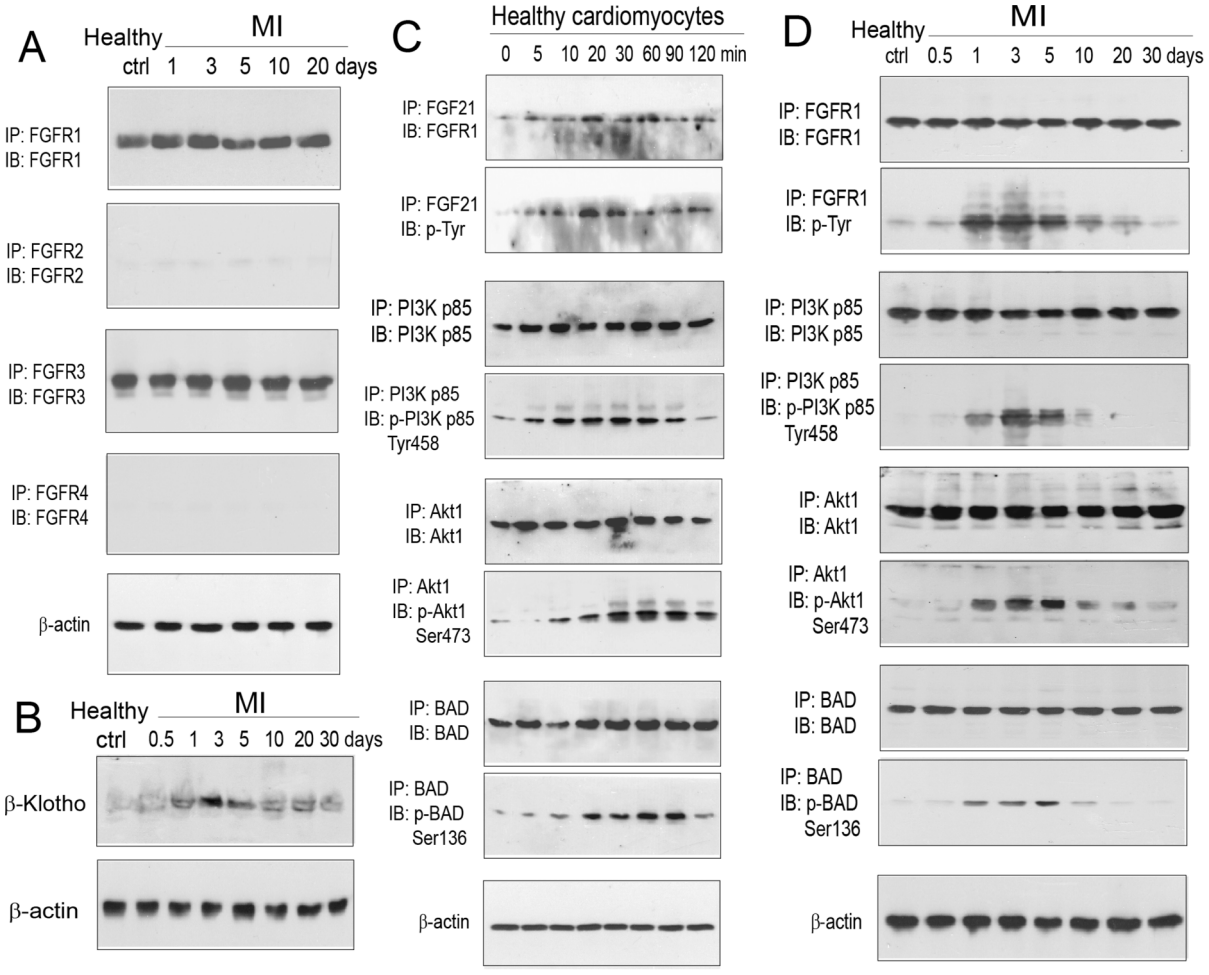
Identification of the FGF21 receptor FGFR1, evaluation of FGF21 binding to FGFR1, and assessment of FGF21 influence on the phosphorylation of FGFR1, PI3K p85, Akt1, and BAD in cardiomyocytes. (A) Relative expression of FGFR1, FGFR2, FGFR3, and FGFR4 in cardiomyocytes isolated from healthy and myocardial ischemic wild-type mice. (B) Relative expression of β-Klotho in cardiomyocytes isolated from healthy and myocardial ischemic wild-type mice. (C) Co-immunoprecipitation of FGFR1 with FGF21 and phosphorylation of FGFR1, PI3K p85, Akt1, and BAD in the presence of FGF21 in freshly isolated cardiomyocytes from healthy wild-type mice *in vitro*. Note that FGFR3 was not able to co-immunoprecipitate with FGF21. (D) Time-dependent changes in the relative expression and phosphorylation of FGFR1, PI3K p85, Akt1, and BAD in cardiomyocytes from the ischemic myocardium of wild-type mice. Note that cropped gel images are used in this figure and the gels were run under the same experimental conditions.

As FGF21 interacts with all known protein tyrosine kinase-coupled FGFRs, including FGFR1, FGFR2, FGFR3, and FGFR4^32^,^36^,^37^, we tested the expression of these FGFRs in cardiomyocytes from wild-type mice with and without myocardial ischemia/reperfusion injury. As shown in Fig. 2A, FGFR1 and FGFR3 were expressed in cardiomyocytes from healthy and myocardial ischemic mice, whereas the expression of FGFR2 and FGFR4 was almost undetectable. The expression level of FGFR1 and FGFR3 was relatively constant from healthy to injured cardiomyocytes through a 10-day observation period. Further tests demonstrated that the FGFR cofactor β-Klotho was expressed at a basal level in the cardiomyocyte of healthy mice, and increased in expression from 1 to 5 days and returned to about the basal level at 10 days following myocardial injury (Fig. 2B). These observations suggested that FGFR1 and FGFR3 were candidate receptors for FGF21, and β-Klotho was possibly involved in FGF21–FGFR interaction in myocardial ischemia/reperfusion injury.

### FGF21–FGFR1 interaction and FGFR1 phosphorylation *in vitro*

To identify a FGF21 receptor, we tested FGF21 binding to FGFR1 or FGFR3 in cardiomyocytes *in vitro*. Cardiomyocytes freshly isolated from healthy wild-type mice were incubated in the presence of recombinant mouse FGF21 (50 ng/ml) *in vitro*, and FGF21 binding to FGFR1 or FGFR3 was tested by co-immunoprecipitation and immunoblot analyses. As shown in Fig. 2C, FGFR1, but not FGFR3, was co-immunoprecipitated with FGF21 from 5 to 120 min following FGF21 treatment. This process was accompanied by tyrosine phosphorylation of FGFR1. These observations suggested FGFR1 as a primary receptor mediating FGF21 signaling in the mouse cardiomyocyte.

### Phosphorylation of PI3K p85, Akt1, and BAD in the presence of FGF21 *in vitro*

As a FGFR potentially activates the PI3K–Akt1 signaling pathway to mediate cell survival, we tested whether FGF21 caused phosphorylation of PI3K p85 and Akt1 as well as a downstream molecule BAD in cardiomyocytes *in vitro*. FGF21 treatment induced phosphorylation of PI3K p85 on Tyr 458, Akt1 on Ser 473, and BAD on Ser 136 in freshly isolated cardiomyocytes (Fig. 2C). The phosphorylation time course of these molecules was similar to that of FGFR1 (Fig. 2C). These observations suggested a potential signaling pathway for FGF21-mediated myocardial protection. 

### Phosphorylation of FGFR1, PI3K p85, Akt1, and BAD in ischemic cardiomyocytes *in vivo*

To demonstrate the involvement of FGFR1 as well as its potential downstream signaling molecules PI3K, Akt1, and BAD in myocardial protection, we tested the relative expression and phosphorylation of these molecules in cardiomyocytes isolated from the ischemic myocardium of wild-type mice. FGFR1 was expressed at a relatively constant level from 0.5 to 30 days, but exhibited a transient increase in tyrosine phosphorylation from 1 to 5 days post myocardial ischemia (Fig. 2D). Similarly, PI3K p85, Akt1, and BAD did not show noticeable changes in relative expression, but exhibited transient phosphorylation on Tyr 458, Ser 473, and Ser 136, respectively, with a time course consistent with that of FGFR1 phosphorylation (Fig. 2D). This time course also coincided with that of FGF21 expression in the liver and adipose tissue as shown in ref. 24 and Fig. 1B, respectively. These observations supported the involvement of FGFR1, PI3K, Akt1, and BAD in FGF21-mediated myocardial protection.

### Role of β-Klotho in mediating FGF21-FGFR1 interaction and FGFR1 phosphorylation

**Figure 3:**
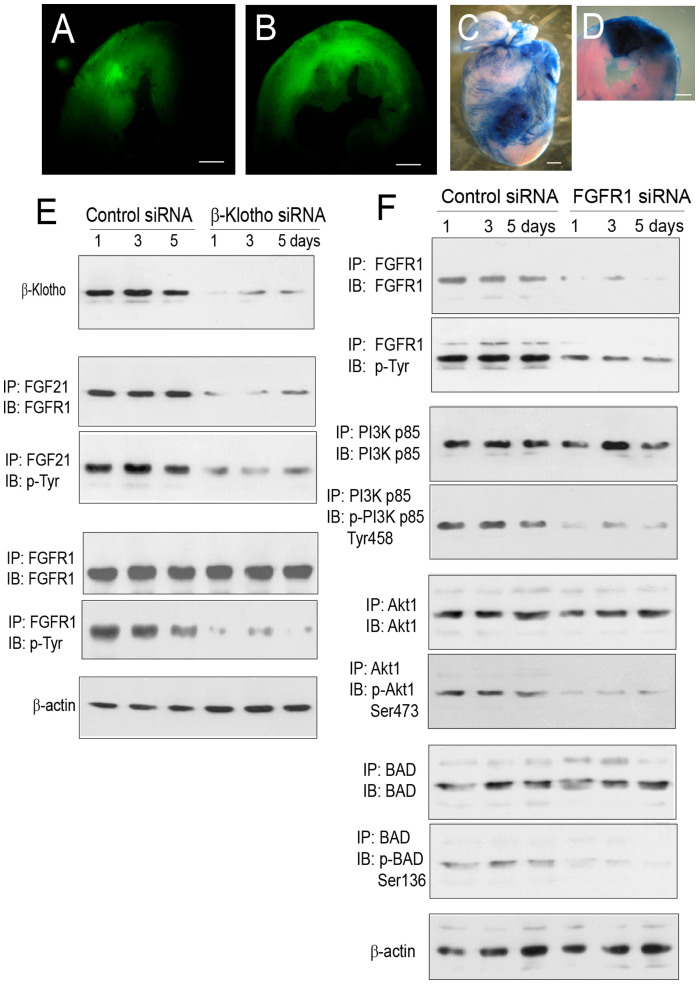
Influence of siRNA-mediated gene silencing on β-Klotho and FGFR1 signaling in cardiomyocytes. (A, B) Fluorescence micrographs of left ventricular (LV) sections showing the distribution and coverage of a FITC-conjugated control siRNA in the myocardium at 1 hr following a single injection (panel A) or 6 equally spaced injections (5 μg/ml, 10 μl for each injection, ~0.5 mm in depth, ~2 mm apart, 2 columns) (panel B) into the left ventricular wall of a beating heart by thoracotomy. Scale bars: 1 mm. (C) The anterior view of a mouse heart showing the distribution of Evans blue immediately following a single injection into the left ventricular wall of a beating heart. Scale bar: 1 mm. (D) A LV slice from the heart shown in panel C demonstrating the distribution of Evans blue within the myocardium. Scale bar: 1 mm. (E) Influence of siRNA-mediated β-Klotho gene silencing on the relative expression of β-Klotho, co-immunoprecipitation of FGFR1 with FGF21, and the relative phosphorylation of FGFR1 in cardiomyocytes isolated from wild-type mice with myocardial injection of a β-Klotho or control siRNA. Myocardial ischemia/reperfusion injury was induced at 3 days after siRNA injection. Immunoprecipitation (IP) and immunoblot (IB) tests were carried out at 1, 3, and 5 days after myocardial injury. (F) Influence of siRNA-mediated FGFR1 gene silencing on the relative expression and phosphorylation of FGFR1, PI3K p85, Akt1, and BAD in cardiomyocytes isolated from wild-type mice with myocardial injection of a FGFR1 or control siRNA. The same protocol was used for the induction of myocardial injury and IP/IB tests. Note that cropped gel images are used in this figure and the gels were run under the same experimental conditions.

β-Klotho is a cell membrane protein known to mediate FGF21–FGFR1 interaction in adipocytes^39^,^40^,^41^,^42^, and is a critical factor for FGF21-mediated regulation of metabolic activities *in vivo*^39^. To test the influence of β-Klotho on FGF21–FGFR1 interaction in cardiomyocytes, we suppressed β-Klotho expression by direct injection of a β Klotho siRNA to the left ventricular anterior wall at 6 equally spaced locations (5 μg/ml, 10 μl each location, ~0.5 mm in depth, ~2 mm apart, 2 columns) in wild-type mice by thoracotomy with a scrambled siRNA as a control. Following siRNA injection, the siRNA distribution and coverage in the myocardium were evaluated by a tracing test with a FITC-conjugated control siRNA. A single injection resulted in a siRNA coverage about 2 mm in radius (Fig. 3A), and 6 equally spaced injections resulted in a siRNA coverage about the size of the ischemic myocardium (~4 mm in radius) (Fig. 3B). These observations were confirmed by the Evans blue tracing test (Fig. 3C and D).

At 3 days following β-Klotho or control siRNA administration, myocardial ischemia/reperfusion injury was induced by 30-min coronary artery ligation. Cardiomyocytes were isolated from the siRNA-covered ischemic myocardium at 1, 3, and 5 days post myocardial injury. β-Klotho siRNA treatment suppressed the expression of β-Klotho in cardiomyocytes (Fig. 3E). The level of FGFR1 co-immunoprecipitation with FGF21 was reduced in cardiomyocytes with β-Klotho siRNA treatment compared to that with control siRNA treatment (Fig. 3E). Furthermore, the relative phosphorylation of FGFR1, while enhanced in the presence of FGF21, was reduced in β-Klotho siRNA-treated cardiomyocytes (Fig. 3E). These observations supported the role of β-Klotho in mediating FGF21–FGFR1 interaction in the ischemic cardiomyocytes.

### Role of FGFR1 in regulating phosphorylation of PI3K p85, Akt1, and BAD

To test whether FGFR1 induces phosphorylation of PI3K p85, Akt1, and BAD *in vivo*, we suppressed FGFR1 expression by direct injection of FGFR1 siRNA to the left ventricular anterior wall of wild-type mice as descried above with a scrambled siRNA as a control and induced myocardial ischemia/reperfusion injury at 3 days after siRNA administration. At 1, 3, and 5 days following myocardial injury, the relative expression and phosphorylation of FGFR1 were reduced in cardiomyocytes with FGFR1 siRNA treatment compared to that with control siRNA treatment (Fig. 3F). When cardiomyocytes from the siRNA-covered ischemic myocardium were incubated in the presence of recombinant mouse FGF21 (50 ng/ml) for 20 min, a procedure known to cause phosphorylation of FGFR1, PI3K p85, Akt1, and BAD (Fig. 2C), the relative phosphorylation of PI3K p85 on Tyr458, Akt1 on Ser473, and BAD on Ser136 in FGFR1 siRNA-treated cardiomyocytes was reduced compared to that in control siRNA-treated cardiomyocytes, although the relative expression of these molecules (FGFR1, PI3K p85, Akt1, and BAD) remained unchanged (Fig. 3F). These observations supported the role of FGFR1 in regulating the phosphorylation of PI3K p85, Akt1, and BAD in cardiomyocytes.

### Attenuation of caspase 3 activity by FGF21

**Figure 4:**
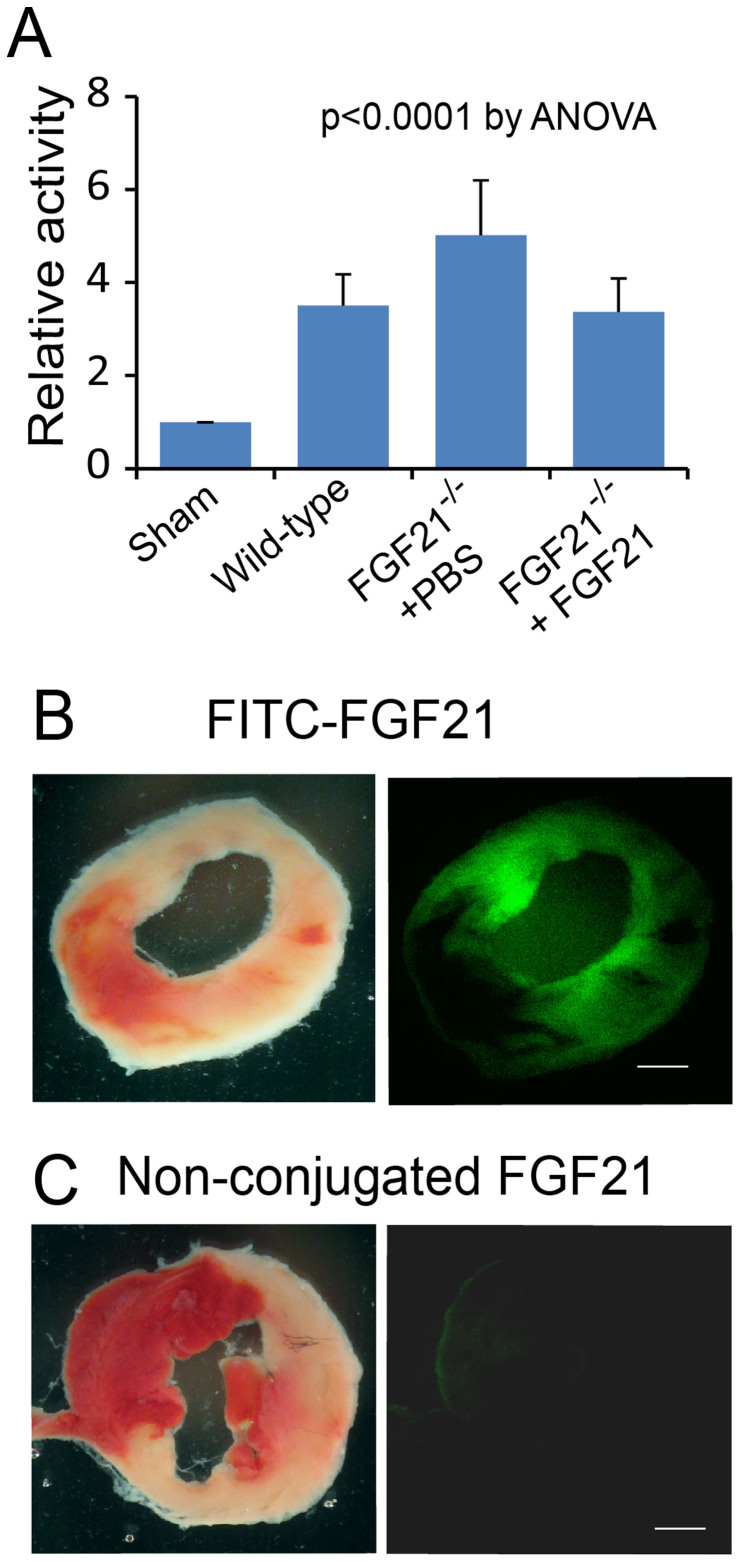
Influence of FGF21 on caspase 3 activity in ischemic cardiomyocytes. (A) Relative activity of caspase 3 in the ischemic cardiomyocytes at 24 hrs, calculated in reference to the relative activity of caspase 3 in the sham control cardiomyocytes. Means and SDs are presented (n = 8). The P value was estimated by ANOVA among all groups. The post-hoc pairwise multiple comparisons P values are presented in Supplementary Table 1. (B) Micrographs of a left ventricular slice showing myocardial infarcts by TTC-staining (left panel) and the presence of FITC-FGF21 within the ischemic myocardium (right panel). Note that FITC-FGF21 was administered intravenously at 24 hrs after myocardial injury and tested at 1 hr after administration. Red: Intact myocardium. White: Infarcts. Green: FITC-FGF21. Scale bar: 1 mm. (C) Micrographs of a left ventricular slice from a mouse with intravenous injection of non-conjugated FGF21 showing the absence of fluorescence in the ischemic myocardium (the injection and testing protocol was the same as that in panel B). Left panel: TTC staining. Right panel: fluorescence micrograph. Scale bar: 1 mm.

Activation of caspase 3 is a key process of apoptosis^52^,^53^. We evaluated the relative activity of caspase 3 in sham control and ischemic cardiomyocytes from wild-type and FGF21^−/−^ mice at 24 hrs, a time chosen because of the presence of maximal cell death as tested by the TUNEL assay^24^. Myocardial ischemia/reperfusion injury caused a significant increase in the relative activity of caspase 3 in wild-type cardiomyocytes. FGF21 deficiency further promoted activation of caspase 3, whereas administration of recombinant FGF21 significantly reduced the relative activity of caspase 3 in cardiomyocytes (Fig. 4A). The accessibility of FGF21 to the ischemic myocardium was confirmed by the FITC-FGF21 tracing test (Fig. 4B and 4C). These investigations suggested an involvement of caspase 3 in myocardial injury and a role for FGF21 in suppressing caspase 3 activation.

### Cardioprotective action of FGF21

**Figure 5:**
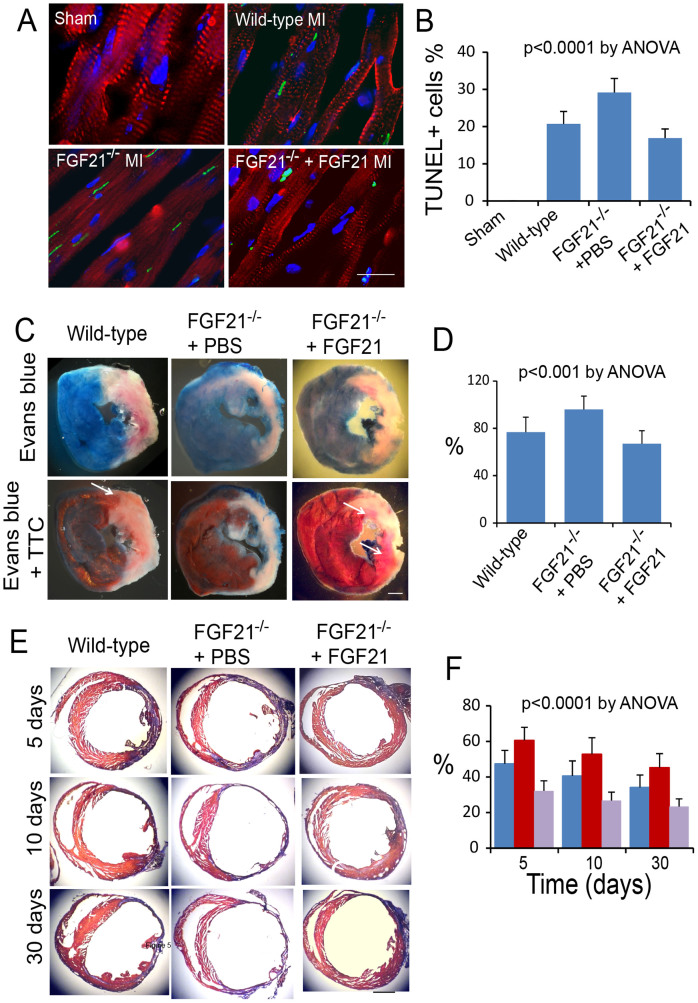
Cardioprotective action of FGF21 in myocardial ischemia/reperfusion injury. (A) Immunofluorescence micrographs showing cells undergoing DNA fragmentation in the ischemic myocardium by the TUNEL assay. Red: Cardiac troponin I. Green: TUNEL-positive cell nuclei. Blue: Cell nuclei. Scale: 10 μm. (B) Graphic representation of the fraction of TUNEL-positive cell nuclei in the ischemic myocardium calculated in reference to the total cell nuclei. Means and SDs are presented (n = 8). The P value was estimated by ANOVA among all groups. The post-hoc pairwise multiple comparisons P values are presented in Supplementary Table 2. (C) Left ventricular slices from wild-type mice and FGF21^−/−^ mice with administration of PBS or recombinant FGF21 at 24 hrs post myocardial ischemia/reperfusion injury, showing the influence of FGF21 on the fraction of acute myocardial infarcts (by the TTC assay) in reference to the area at risk (by the Evans blue assay). Note that the left ventricular wall thickness is thinner in FGF21^−/−^ mice with PBS administration than that in wild-type mice and FGF21^−/−^ mice with FGF21 administration. Arrows: TTC-positive (red) myocardium within the area at risk. Scale: 1 mm. (D) Graphic representation of the influence of FGF21 on the fraction of acute myocardial infarcts in reference to the area at risk. Means and SDs are presented (n = 8). The P value was estimated by ANOVA among all groups. The post-hoc pairwise multiple comparisons P values are presented in Supplementary Table 3. (E) AZAN-stained left ventricular sections from wild-type mice and FGF21^−/−^ mice with administration of PBS or recombinant FGF21 at 5, 10, and 30 days after myocardial ischemia/reperfusion injury. Red: Intact myocardium. Blue: Myocardial infarcts and fibrous tissue. Scale bar: 1 mm. (F) Graphic representation of the fraction of myocardial infarcts in wild-type mice (blue) and FGF21^−/−^ mice with administration of PBS (red) or recombinant FGF21 (purple) at 5, 10, and 30 days after myocardial injury. Means and SDs are presented (n = 6). The P value was estimated by ANOVA and is <0.0001 for both time- and treatment-based comparisons. The post-hoc pairwise multiple comparisons P values are presented in Supplementary Table 4.

We evaluated the cardioprotective action of FGF21 based on the TUNEL index in wild-type and FGF21^−/−^ mice at 24 hrs of myocardial ischemia/reperfusion injury. This observation time was chosen because of the presence of maximal cell death as tested in a previous study^24^. FGF21 deficiency caused a significant increase in the TUNEL index (29+/−4, n = 8, p < 0.001) compared to the wild-type control (21+/−3, n = 8), whereas administration of recombinant FGF21 reduced the TUNEL index in FGF21^−/−^ mice (17+/−3, n = 8, p < 0.001; Fig. 5A and 5B). These observations suggested that FGF21 contributed to myocardial protection in myocardial ischemia/reperfusion injury.

We further evaluated the short-term (24 hrs) effect of FGF21 on the degree of myocardial infarction in reference to the area at risk (Fig. 5C and 5D). At 24 hrs, the fraction of the area at risk for wild-type mice was 61.7+/−10.7% (n = 8) in reference to the left ventricular wall volume below the coronary artery ligation and that for FGF21^−/−^ mice with PBS or recombinant FGF21 administration was 58.4+/−8.3 and 56.6+/−7.9%, respectively (n = 8 for both). No significant difference was detected between the three groups by one-way ANOVA (p > 0.1), suggesting that a similar area at risk was established. The fraction of myocardial infarcts by the TTC assay in reference to the area at risk was 76+/−13% (n = 8) in wild-type mice. This fraction was significantly increased in FGF21^−/−^ mice (95+/−11, n = 8, p < 0.05) compared to that in wild-type mice (Fig. 5C and 5D). Administration of recombinant FGF21 (50 ng/gm for each dose, 2 doses per day with a 12 hr interval) reversed the change due to FGF21 deficiency (67+/−11%, n = 8, p < 0.0001).

The long-term cardioprotective effect of FGF21 is sown in Fig. 5E, 5F, and Table 1. FGF21^−/−^ mice exhibited a significant increase in the fraction of myocardial infarcts compared to wild-type mice at 5, 10, and 30 days following myocardial ischemia/reperfusion injury. Administration of recombinant FGF21 (50 ng/gm for each dose, 2 doses per day with a 12 hr interval for a total period of 3 days) to FGF21^−/−^ mice caused a significant drop in the fraction of myocardial infarcts compared to PBS administration (Fig. 5E and 5F). These observations supported the long-term cardioprotective effect of FGF21 in myocardial ischemia/reperfusion injury. This long-term beneficial effect was probably a result from the acute phase protective action of FGF21.

### Improvement of left ventricular function by FGF21

**Figure 6:**
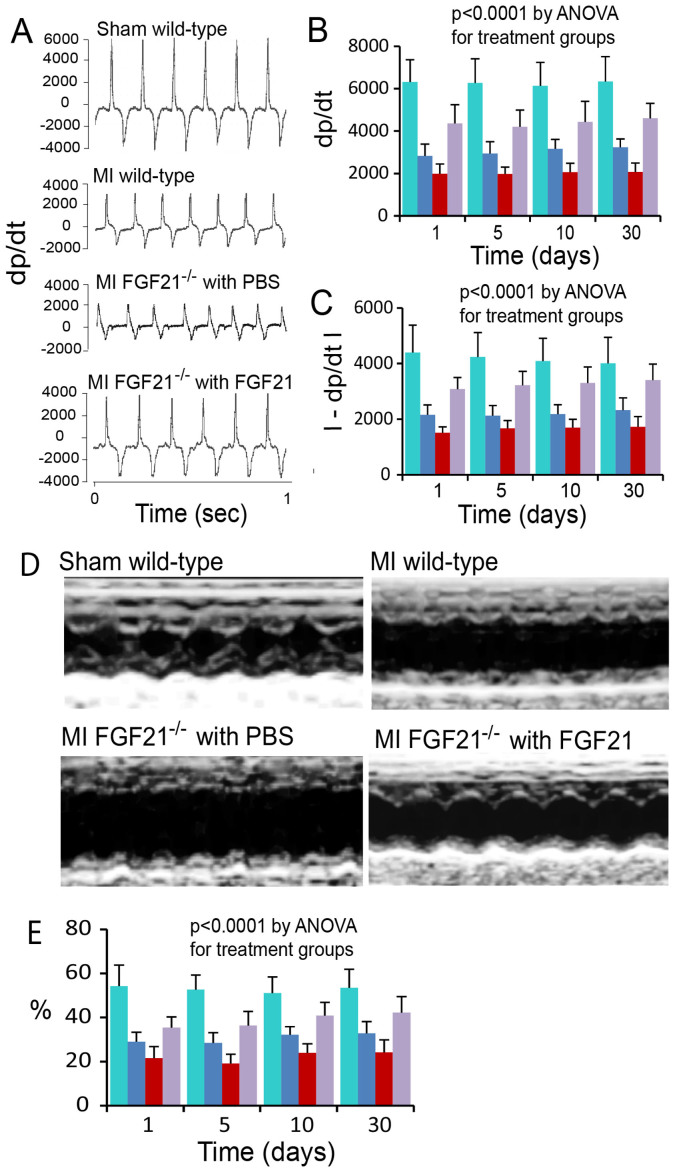
Effect of FGF21 on the left ventricular (LV) function in myocardial ischemia/reperfusion injury. (A) LV dp/dt and −dp/dt recorded from sham-operated and myocardial ischemic wild-type mice and myocardial ischemic FGF21^−/−^ mice with administration of PBS or recombinant mouse FGF21 at day 5 following myocardial injury. (B, C) Graphic representation of the effect of FGF21 on the LV dp/dt and −dp/dt (absolute value) at 1, 5, 10, and 30 days following myocardial injury. Green and blue bars: Wild-type mice with sham operation or myocardial injury, respectively. Red and purple bars: Myocardial ischemic FGF21^−/−^ mice with PBS or FGF21 administration, respectively. Means and SDs are presented (n = 6). The P value was estimated by ANOVA and is <0.0001 only for comparisons in dp/dt and −dp/dt among treatment groups including wild-type sham, wild-type MI, FGF21^−/−^ MI with PBS administration, and FGF21^−/−^ MI with FGF21 administration. P > 0.05 for comparisons in dp/dt and −dp/dt among different times including 1, 5, 10, and 30 days by ANOVA. The post-hoc pairwise multiple comparisons P values are presented in Supplementary Table 5. (D) Echocardiographs of the left ventricle of sham-operated and myocardial ischemic wild-type mice and myocardial ischemic FGF21^−/−^ mice with PBS or FGF21 administration at 5 days following myocardial injury. (E) Fractional shortening of the left ventricle of sham-operated (green) or myocardial ischemic (blue) wild-type mice and myocardial ischemic FGF21^−/−^ mice with administration of PBS (red) or FGF21 (purple). Means and SDs are presented (n = 6). The P value was estimated by ANOVA and is <0.0001 only for comparisons among treatment groups including wild-type sham, wild-type MI, FGF21^−/−^ MI with PBS administration, and FGF21^−/−^ MI with FGF21 administration. P > 0.05 for comparisons among different times including 1, 5, 10, and 30 days by ANOVA. The post-hoc pairwise multiple comparisons P values are presented in Supplementary Table 6.

We evaluated the influence of FGF21 on the left ventricular (LV) dp/dt and fractional shortening in wild-type and FGF21^−/−^ mice with myocardial ischemia/reperfusion injury. While both wild-type and FGF21^−/−^ mice exhibited a reduction in the LV dp/dt and −dp/dt (absolute value) in myocardial injury compared to sham-operated mice, FGF21^−/−^ mice showed significantly lower levels compared to wild-type mice (Fig. 6A–C, Table 2). Administration of recombinant FGF21 significantly improved the LV dp/dt and −dp/dt (absolute value) in FGF21^−/−^ mice with myocardial injury in reference to PBS administration (Fig. 6A–C, Table 2). A similar pattern of change was observed for the fractional shortening of the ischemic left ventricle. FGF21^−/−^ mice with PBS administration exhibited a significantly lower LV fractional shortening compared to wild-type mice (Fig. 6D and 6E, Table 2). FGF21 administration reversed the change due to FGF21 deficiency (Fig. 6D and 6E, Table 2). The heart beating rate, a parameter potentially influencing the LV dp/dt and fractional shortening, varied between the tested groups, but did not show a significant difference (p > 0.1, n = 6, see Table 2). These observations supported the cardioprotective action of FGF21 in myocardial ischemia/reperfusion injury.

### Role of FGFR1, β-Klotho, PI3K, and Akt1 in regulating FGF21-based myocardial protection

**Figure 7:**
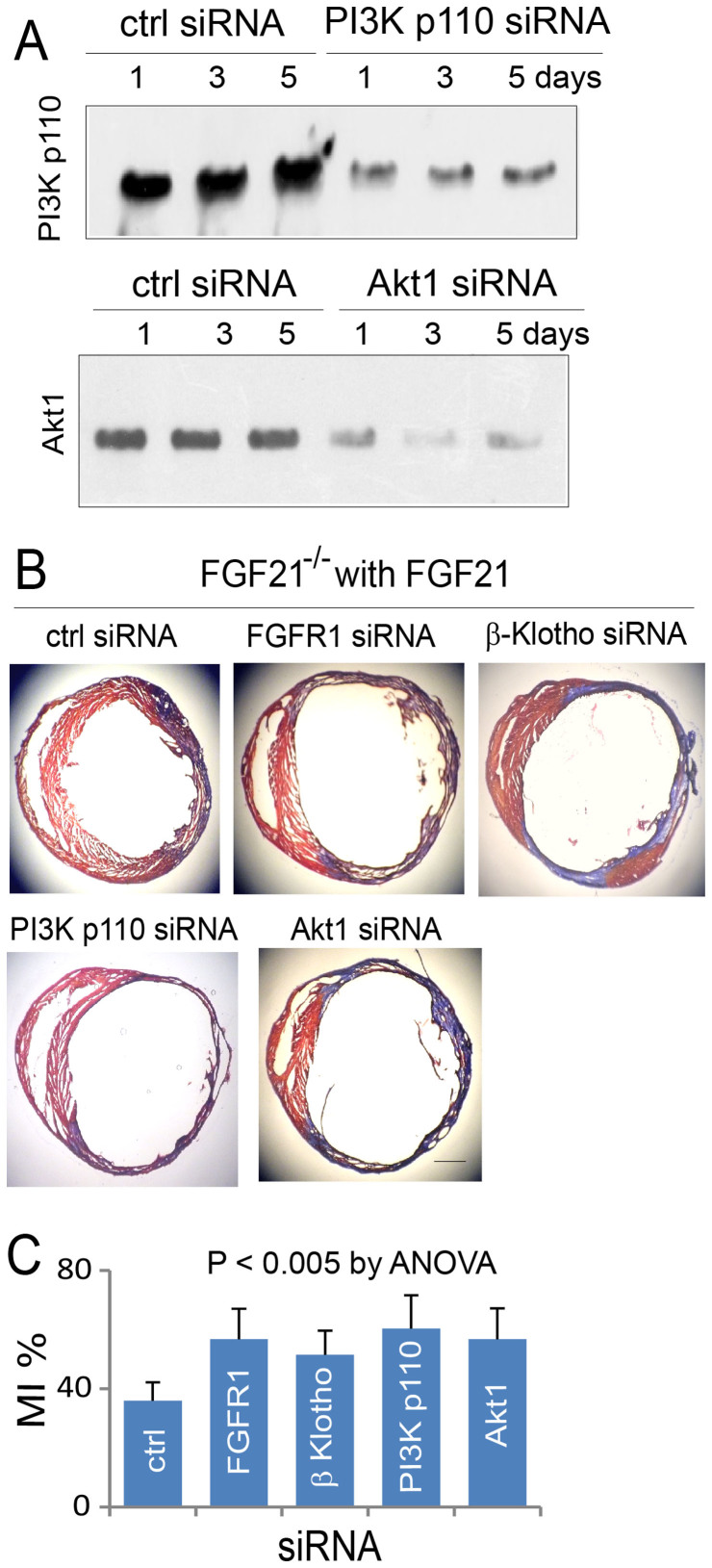
Influence of siRNA-mediated FGFR1, β-Klotho, PI3K p110, or Akt1 gene silencing on the fraction of myocardial infarcts. (A) Relative expression of PI3K p110 and Akt1 in cardiomyocytes at 1, 3, and 5 days after myocardial injury (note that a PI3K p110, Akt1, or control siRNA was injected into the left ventricular anterior wall at 3 days prior to myocardial injury). The effect of siRNA on FGFR1 and β-Klotho expression is presented in Fig. 3. Note that cropped gel images are used in this figure and the gels were run under the same experimental conditions. (B) AZAN-stained left ventricular sections from FGF21^−/−^ mice administered with a control, FGFR1, β-Klotho, PI3K p110, or Akt1 siRNA (siRNA injected into the myocardium at 3 days prior to myocardial injury and specimens collected at 5 days following myocardial ischemia/reperfusion injury). These mice were administered with recombinant FGF21 by intravenous injection immediately following myocardial injury for 3 days with a 12 hr interval to establish FGF21-based myocardial protection. Scale bar: 1 mm. (C) Graphic representation of the influence of siRNA-mediated FGFR1, β-Klotho, PI3K p110, or Akt1 gene silencing on the fraction of myocardial infarcts at day 5. Means and SDs are presented (n = 7). The P value was estimated by ANOVA among all groups. The post-hoc pairwise multiple comparisons P values are presented in Supplementary Table 7.

As the aforementioned observations supported the involvement of FGFR1, β-Klotho, PI3K, and Akt1 in FGF21 signaling, we confirmed the role of these molecules in the regulation of FGF21-based myocardial protection by siRNA modulation in FGF21^−/−^ mice with administration of recombinant FGF21 (Fig. 7), a model showing the cardioprotective effect of FGF21. A FGFR1, β-Klotho, PI3K p110, Akt1, or control siRNA was directly injected to the left ventricular anterior wall of FGF21^−/−^ mice by the 6-injection approach, myocardial ischemia/reperfusion injury was induced 3 days following siRNA administration, and the fraction of myocardial infarcts was measured and analyzed at 5 days following myocardial injury. Administration of a β-Klotho, FGFR1, PI3K p110, or Akt1 siRNA suppressed the relative expression of the cognate target protein by 73+/−12, 78+/−15, 75+/−16, or 69+/−11% (n = 6), respectively, at day 4 after siRNA administration (or day 1 after myocardial injury that was induced at day 3 following siRNA administration). Treatment with a FGFR1, β-Klotho, PI3K p110, or Akt1 siRNA suppressed the cardioprotective effect of FGF21 in reference to the control siRNA treatment (Fig. 7B and 7C). A global ANOVA analysis showed a significant difference among the groups with control and specific siRNA treatments (P < 0.005, n = 7 each group, Fig. 7C). In terms of individual siRNA treatment, administration of a FGFR1, PI3K p110, or Akt1 siRNA caused significant intensification of myocardial infarction. While a β-Klotho siRNA treatment induced an increase in myocardial infarction, the change did not reach a statistically significant level based on the conservative P values from the multiple comparisons test. However, the Student t-test showed that the change due to a β-Klotho siRNA treatment in reference to a control siRNA treatment was statistically significant (P < 0.01). These observations suggest a role for FGFR1, β-Klotho, PI3K, and Akt1 in mediating FGF21-based myocardial protection.

## Discussion

An important discovery from this investigation, along with our recent studies^24^, was that myocardial ischemia-induced FGF21 from the liver and adipose tissue was involved in endocrine myocardial protection, and prompt administration of this protein significantly mitigated myocardial infarction. While our recent studies demonstrated the expression of FGF21 in the liver in response to experimental myocardial ischemia and the cardioprotective role of FGF21 during the acute phase of myocardial injury (24 hrs)^24^, the present investigation demonstrated FGF21 upregulation in not only the liver, but also the adipose tissue. This investigation also showed the long-term cardioprotective effect of FGF21 in myocardial ischemia/reperfusion injury. FGF21 has been known to regulate glucose and lipid metabolism^30^,^31^,^32^,^33^,^34^,^35^. The concept of FGF21 mediation of myocardial protection is novel.

Several lines of evidence supported the cardioprotective action of FGF21. First, FGF21 was upregulated in hepatic cells and adipocytes in response to myocardial ischemia/reperfusion injury. Compared to the sham control level, the transcription of the hepatic FGF21 gene was increased by about 11 times in myocardial ischemia^21^, accompanied by protein-level FGF21 upregulation in the hepatocytes and adipocytes in response to myocardial injury. These changes were associated with elevation of serum FGF21^24^, suggesting an endocrine mechanism for myocardial protection. Second, FGF21^−/−^ mice exhibited a significant increase in caspase 3 activity, cell death, and myocardial infarction accompanied by significant deterioration of the left ventricular function compared to wild-type mice, whereas administration of recombinant FGF21 reversed the changes due to FGF21 deficiency. These observations supported the cardioprotective action of FGF21 in myocardial ischemia/reperfusion injury. The potential impact of this investigation is that FGF21 may be considered a therapeutic agent for myocardial protection.

An important concept from this investigation is that myocardial protection involves endocrine mechanisms. Previous investigations have demonstrated autocrine/paracrine cardioprotective processes within the ischemic myocardium, including release of adenosine^1^,^2^, opioids^3^,^4^, and bradykinin^5^,^6^, upregulation of VEGF^7^,^8^,^9^,^10^, and activation of cardiac resident stem cells^11^,^12^,^13^,^14^. Here, we demonstrated that the liver and adipose tissue were activated in myocardial ischemia/reperfusion injury to upregulate and release FGF21 for myocardial protection. Such an endocrine response is likely established to boost the protection of the ischemic myocardium, a vital structure with a limited capacity of protection.

A fundamental question is how FGF21 contributes to myocardial protection. This investigation demonstrated that FGF21 protected the ischemic cardiomyocytes via interaction with FGFR1, which activated the PI3K–Akt1 signaling pathway and induced phosphorylation of the cell death inducer BAD, thereby reducing apoptosis and supporting cardiomyocyte survival. It has been documented that the phosphorylation state of BAD determines its activity as a cell death inducer^8^,^48^. When dephosphorylated, BAD promotes cell apoptosis by sequestering the anti-apoptotic proteins Bcl-2 and/or Bcl-XL, rendering the pro-apoptotic factors BAX and BAK more active^48^,^54^,^55^. BAX and BAK induce pore formation in the mitochondrial outer membrane, allowing cytochrome C to escape from the mitochondria into the cytosol. Cytochrome C binds and activates apoptotic protease activating factor 1 (Apaf1), resulting in activation of caspase 9. Caspase 9 in turn activates caspase 3, an effector proteinase that degrades proteins and causes apoptosis^52^,^53^. When phosphorylated, BAD loses its power for promoting cell apoptosis by releasing the anti-apoptotic proteins Bcl-2 and Bcl-XL, which become active to suppress cell apoptosis^48^. The present investigation suggested BAD phosphorylation as a process mediating the cardioprotective action of the FGF21–FGFR1/β-Klotho–PI3K–Akt1 signaling network.

To date, numbers of innate cardioprotective factors have been identified, including adenosine^1^,^2^, opioids^3^,^4^, bradykinin^5^,^6^, and VEGF^7^,^8^,^9^,^10^, which are released from injured and activated cardiac cells in response to myocardial ischemia. An interesting observation is that most of these factors activate PI3K and Akt1 for myocardial protection^8^. As shown in the present study, PI3K and Akt1 were also involved in FGF21-mediated myocardial protection. These investigations suggest PI3K and Akt1 as key molecules converging signals from various cell survival-supporting ligands to a common pathway for myocardial protection. The expression of abundant cell survival ligands is possibly a mechanism evolved to maximize the cardioprotective action in response to ischemic injury.

In this investigation, we used siRNAs to suppress the expression of selected signaling molecules in cardiomyocytes by myocardial injection. A technical concern is whether this approach can be used to establish a sufficiently large siRNA coverage with uniform siRNA distribution in the ischemic myocardium for effective gene silencing. We carried out a FITC-siRNA tracing test to address this concern and demonstrated that a single siRNA injection resulted in a uniform siRNA coverage about 2 mm in radius and 6 equally spaced injections (~0.5 mm in depth, ~2 mm apart, 2 columns) resulted in a uniform siRNA coverage about the size of the ischemic myocardium (~4 mm in radius). These observations were further confirmed by the Evans blue tracing test. More importantly, the relative expression of the target proteins was reduced by about 70% in cardiomyocytes at 4 days following siRNA administration. These observations suggest that direct myocardial siRNA injection is a reliable and effective method for gene silencing in cardiomyocytes *in vivo*. Compared to the transgenic approach, it is easier to silence the expression of a target gene by the siRNA approach with an acceptable efficacy. Furthermore, the siRNA approach can be used to modulate multiple genes simultaneously in a single animal. However, intra-myocardial administration requires thoracotomy, an approach causing injury to the animal.

## Methods

### Myocardial ischemia/reperfusion injury

We induced myocardial ischemia/reperfusion injury in FGF21^−/−^ (Taconic Farms) and wild-type mice by 30-min occlusion of the left anterior descending (LAD) coronary artery. The FGF21^−/−^ model was generated as described^56^,^57^. Mice were anesthetized by intraperitoneal injection of ketamine (100 mg/kg)/xylazine (10 mg/kg) and ventilated via the trachea by using a rodent respirator. Intercostal thoracotomy was carried out and the LAD coronary artery was occluded for 30 min by suture ligation immediately above the second diagonal branch^24^. Gender- and body weight-matched mice with sham operation were used as controls. Experimental observations were carried out at 1, 5, 10, and 30 days or other times, depending on the nature of the test, following myocardial injury with a sample size of 6–8 mice determined by statistical power analyses.

In randomly selected FGF21^−/−^ mice, recombinant mouse FGF21 was administered intravenously at 50 ng/g of body weight immediately after myocardial injury followed by additional administrations every 12 hrs for 3 days with PBS as a control. The 3-day FGF21 administration strategy was based on the observation that cell death in the ischemic myocardium occurred primarily during the first 3 days^24^. The effect of FGF21 administration on myocardial infarction was tested at 1, 5, 10, and 30 days in reference to that of PBS administration. Experimental procedures were approved by the Northwestern University Institutional Animal Care and Use Committee.

### FGF21 expression in selected organs and cell types

We have shown that hepatic cells upregulate FGF21 in response to myocardial ischemia^24^. It is possible that other cell types also express FGF21 in myocardial ischemia. In this investigation, we tested FGF21 expression in selected organs and tissues including the brain, ischemic myocardium, lung, liver, pancreas, spleen, kidney, small intestine, adipose tissue, and skeletal muscle at 1 day following myocardial injury, a time with FGF21 upregulation in the liver^24^. The selected organs were perfused with PBS by arterial cannulation to remove the circulating blood. FGF21 expression was assessed by immunoprecipitation and immunoblot analyses as described^58^,^59^. β-actin from the same protein samples was tested and used as a control. As the liver and adipose tissue expressed FGF21 in myocardial ischemia/reperfusion injury, we further tested FGF21 expression in hepatocytes and adipocytes isolated by collagenase treatment and centrifugation as described^24^,^60^.

### Expression and phosphorylation of signaling molecules

As FGF21 potentially interacts with FGFRs that may activate the PI3K-Akt1 signaling pathway, we tested in cardiomyocytes the expression and phosphorylation of FGFR1, FGFR2, FGFR3, FGFR4, PI3K p85, and Akt1, as well as a potential downstream molecule BAD. Cardiomyocytes were isolated from the left ventricular portion below the second diagonal bifurcation of healthy, sham-operated, and myocardial ischemic mice by collagenase treatment (0.25%, perfused through the aorta at 100 mm Hg pressure in a 37°C incubator for 20 min) and centrifugation (25 g for 3 min) as described^61^. The isolated cardiomyocytes were lysed in RIPA buffer and used for immunoprecipitation and immunoblot analyses. Antibodies for FGFRs, phosphotyrosine, PI3K p85, phospho-PI3K p85 (Tyr458), Akt1, phospho-Akt1 (Ser473), BAD, and phospho-BAD (Ser136) were from Cell Signaling Biotechnology. We also tested β-Klotho expression in cardiomyocytes by using an anti β-Klotho antibody from R&D Systems. β-actin from the same protein samples was tested and used as a control.

### Evaluation of FGF21 access to ischemic myocardium

This investigation was designed to assess the cardioprotective action of endogenous endocrine or intravenously administered FGF21. A critical question is whether FGF21 can access the ischemic myocardium. We used a FITC-FGF21 tracing method to address this question. FITC-conjugated recombinant mouse FGF21 (50 ng/g of body weight) was administered to mice intravenously at 24 hrs following myocardial ischemia/reperfusion injury. At 1 hr following FITC-FGF21 administration, the vascular system was perfused with PBS via carotid artery cannulation to remove the circulating FITC-FGF21 while the vena cava was cut for draining blood. The heart was removed, cut into 1 mm slices, and used to identify myocardial infarcts by the 2,3,5-triphenyltetrazolium chloride (TTC) assay and visualize FITC-FGF21 in the ischemic myocardium by fluorescence microscopy.

### Myocardial administration of siRNA

We used a siRNA gene silencing approach to evaluate the signaling action of FGFR1, β-Klotho, PI3K, and Akt1 in cardiomyocytes *in vivo*. A FGFR1, β-Klotho, PI3K p110, Akt1, or control siRNA (5 μg/ml) was prepared in a transfection medium mixed with a 0.2% liposome transfection reagent (Santa Cruz Biotechnology). Thoracotomy was carried out in anesthetized FGF21^−/−^ mice. The siRNA mix was directly injected into the left ventricular anterior wall, where ischemia/reperfusion injury was to be induced, at 6 equally spaced locations (10 μl each location, ~0.5 mm in depth, ~2 mm apart, 2 columns) using a Hamilton micro-syringe. The thoracic wound was closed and the mouse was allowed to recover. The coverage and distribution of siRNA in the myocardium were tested by using a FITC-siRNA tracing method. Evans blue was injected to the myocardium to confirm the tracing test. Myocardial ischemia/reperfusion injury was induced 3 days after siRNA administration. The effect of siRNA administration on the expression of FGFR1, β-Klotho, PI3K p110, or Akt1 was tested in cardiomyocytes isolated from the siRNA-covered area.

Various experimental strategies were used for different molecules. To assess the influence of FGFR1 gene silencing on signaling events, the expression and phosphorylation of PI3K p85, Akt1, and BAD were tested in cardiomyocytes with FGFR1 or control siRNA administration. To assess the influence of β-Klotho gene silencing on signaling events, FGF21-FGFR1 interaction as well as FGFR1 expression and phosphorylation were tested. The degree of myocardial infarction was evaluated following the administration of each specific siRNA compared to control siRNA administration.

### Caspase 3 activity assay

The relative activity of caspase 3 was measured in sham control and ischemic cardiomyocytes from wild-type and FGF21^−/−^ mice by using a caspase 3 colorimetric activity assay kit (Chemicon International). At 24 hrs following myocardial ischemia/reperfusion injury or sham operation, cardiomyocytes were isolated by collagenase treatment and centrifugation. The relative activity of caspase 3 in sham control and ischemic cardiomyocytes was measured and analyzed as described^62^. The observation time 24 hrs was chosen because maximal cell death was found at this time^24^.

### Assessment of cell death

The TUNEL assay was used to assess cell death in the sham control and ischemic myocardium. At 24 hrs following myocardial ischemia/reperfusion injury or sham operation, the heart of an anesthetized mouse was fixed by arterial perfusion of 4% formaldehyde in PBS. The observation time 24 hrs was chosen because maximal cell death was found at this time^24^. Specimens were collected from the sham control and ischemic myocardium, and cut into 10 μm cryo-sections. At least 6 specimen sections, equally spaced through an ischemic or sham control region, were collected from each mouse for measurement. The TUNEL test was carried out as described^24^ and Hoechst 33258 was used for detecting cell nuclei. TUNEL-positive cell nuclei were measured from 6 randomly selected regions of each specimen section. A TUNEL index was calculated as the percentage of the TUNEL-positive cell nuclei in reference to Hoechst 33258-labeled total cell nuclei.

### Measurement of myocardial infarcts

We measured the area at risk and fraction of myocardial infarcts at 24 hrs by the Evans blue and TTC assay^63^, respectively, as well as the fraction of myocardial infarcts at 5, 10, and 30 days by the AZAN assay^24^. For the Evans blue and TTC assay, a mouse with 24-hr myocardial ischemia/reperfusion injury was anesthetized and ventilated, the heart was exposed by thoracotomy, the LAD coronary artery was re-ligated at the original ligation location, Evans blue (2% in PBS, 10 μl/g of body weight) was rapidly injected into the left ventricle, and the heart was removed at approximately 5 sec following Evans blue injection. The heart was cut into ~1 mm serial transverse sections. The sections were photographed for measurement of the area at risk, incubated in 1% TTC/PBS at 37°C for 30 min, fixed in 4% formaldehyde/PBS for 15 min, and photographed for measurement of myocardial infarcts. The fraction of myocardial infarcts was calculated in reference to the area at risk.

For the AZAN assay, the mouse heart was fixed by arterial perfusion of 4% formaldehyde/PBS at 100 mm Hg pressure at 5, 10, and 30 days following myocardial ischemia/reperfusion injury and cut into 50 μm serial transverse cryo-sections. One of every five specimen sections was collected from the left ventricle (LV) and stained with AZAN reagents. The areas of infarcts/fibrosis (blue in color) and intact myocardium (red) were measured from each section. The volumes of the infarcts/fibrosis and the intact LV myocardium were calculated based on the measured areas and specimen thickness. The fraction of myocardial infarcts/fibrosis was calculated in reference to the LV wall volume below the coronary artery occlusion.

### Left ventricular fractional shortening by echocardiography

We measured the fractional shortening of the left ventricle by echocardiography^24^. Mice were anesthetized by intraperitoneal injection of tribromoethanol (250 mg/kg body weight at concentration of 1.25%). The systolic and diastolic LV diameters were measured at the middle point of the left ventricle by using a SonoScape ultrasound system with a high-resolution matrix probe. The location of the measurement was identified by B mode echocardiography. The fractional shortening of the left ventricle was calculated as [(diastolic diameter – systolic diameter)/diastolic diameter] × 100^24^.

### Left ventricular dp/dt and −dp/dt

We tested the left ventricular dp/dt and −dp/dt based on blood pressure measurements. Mice were anesthetized by intraperitoneal injection of tribromoethanol (250 mg/kg body weight at concentration of 1.25%). A Millar catheter pressure transducer was inserted into the left ventricle via the right carotid artery. The left ventricular pressure was measured and used to generate dp/dt and −dp/dt from mice with sham operation and myocardial ischemia/reperfusion injury^24^.

### Statistics

Means and standard deviations were calculated for all measured parameters. We used the analysis of variance (ANOVA) method for global comparisons among multiple groups. One-way ANOVA was used for analyses involving a single factor such as time, whereas two-way ANOVA was used for analyses involving two factors such as a treatment and time. Post-hoc pairwise multiple comparisons were carried out by the Tukey, Bonferroni, Scheffé, and Fisher's least significant difference methods. The most conservative method was selected and used for difference evaluation. Sample sizes were estimated by power analysis. A difference was considered statistically significant at p < 0.05.

## Author Contributions

S.Q.L. contributed to experimental design, tests, data analyses, and manuscript preparation. D.R. and Y.H.W. contributed to experimental tests and analyses. A.K. contributed to development of the FGF21^−/−^ mouse model and assisted in manuscript preparation. B.Z. and S.M.H. contributed to manuscript revision. Y.C.L. and L.Q.Z. contributed to experimental design. All authors reviewed the manuscript.

## Supplementary Material

Supplementary Information Supplementary Information

## Figures and Tables

**Table 1:** Fractions of myocardial infarcts in reference to the left ventricular volume below the coronary artery ligation at 5, 10, and 30 days

Time (days)	Wild-type (%)	FGF21^−/−^ + PBS (%)	FGF21^−/−^ + FGF21 (%)
5	48 ± 8	61 ± 9	32 ± 5
10	41 ± 7	53 ± 8	27 ± 4
30	34 ± 6	45 ± 7	23 ± 5

Means and SDs are presented.

**Table 2:** Hemodynamic parameters of the left ventricle in mice with sham operation and myocardial ischemia/reperfusion injury

	Wild-type sham			Wild-type MI			FGF21^−/−^ MI + PBS			FGF21^−/−^ MI + FGF21		
Time (days)	HR	dp/dt	FS (%)	HR	dp/dt	FS (%)	HR	dp/dt	FS (%)	HR	dp/dt	FS (%)
1	444 ± 56	6320 ± 1046	54 ± 10	451 ± 69	2827 ± 557	29 ± 4	466 ± 64	1986 ± 463	22 ± 5	447 ± 77	4366 ± 872	35 ± 5
5	438 ± 54	6270 ± 1135	53 ± 7	448 ± 63	2944 ± 554	29 ± 5	460 ± 66	1978 ± 329	19 ± 4	446 ± 59	4202 ± 787	36 ± 6
10	428 ± 59	6138 ± 1097	51 ± 7	436 ± 54	3167 ± 442	32 ± 4	443 ± 40	2060 ± 426	24 ± 4	427 ± 54	4430 ± 968	41 ± 6
30	441 ± 69	6336 ± 1179	54 ± 8	449 ± 63	3234 ± 393	33 ± 5	450 ± 49	2069 ± 427	24 ± 6	434 ± 66	4606 ± 703	42 ± 7

MI: Myocardial ischemia/reperfusion injury. HR: Heart beating rate (beats/min). dp/dt: mm Hg/sec. FS: Fractional shortening. Means and SDs are presented.
