# Supplementary material for: Endocrine Protection of Ischemic Myocardium by FGF21 from the Liver and Adipose Tissue
Source: Sci Rep. 2013 Sep 26;3:2767. doi: 10.1038/srep02767 (PMC3783882; doi:10.1038/srep02767)
Supplement: Supplementary Information [file srep02767-s1.pdf]

Supplementary information

SREP-13-02864A

Endocrine Protection of Ischemic Myocardium by FGF21 from the Liver and Adipose Tissue

Shu Q. Liu[1*], Derek Roberts[1], Alexei Kharitonenkov[2], Brian Zhang[1], Samuel M. Hanson[1], Yan Chun Li[3], Li-Qun Zhang[4], Yu H. Wu[1]

[1]Biomedical Engineering Department, Northwestern University, Evanston, IL 60208, USA.

[2]Diabetes Research, Lilly Research Laboratories, Indianapolis, IN 46285, USA.

[3]Department of Medicine, Division of Biological Sciences, The University of Chicago, Chicago, IL 60637, USA.

[4]Rehabilitation Institute of Chicago, Chicago, IL 60611, USA.

Address for correspondence:

Shu Q. Liu, PhD
Biomedical Engineering Department
Northwestern University
2145 Sheridan Road
Evanston, IL 60208-3107

Phone: 847 491 5745
FAX: 847 491 4928

* Correspondence to [sliu@northwestern.edu]

Supplementary Figures

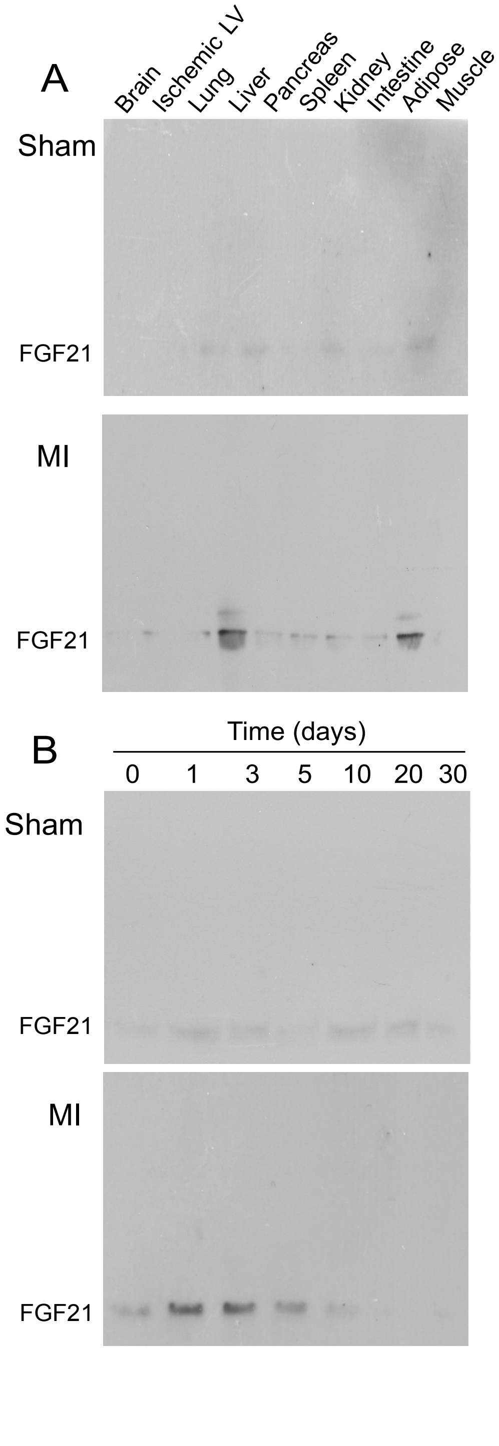

Figure 1. Original immunoblot images for Figure 1 in the main text.

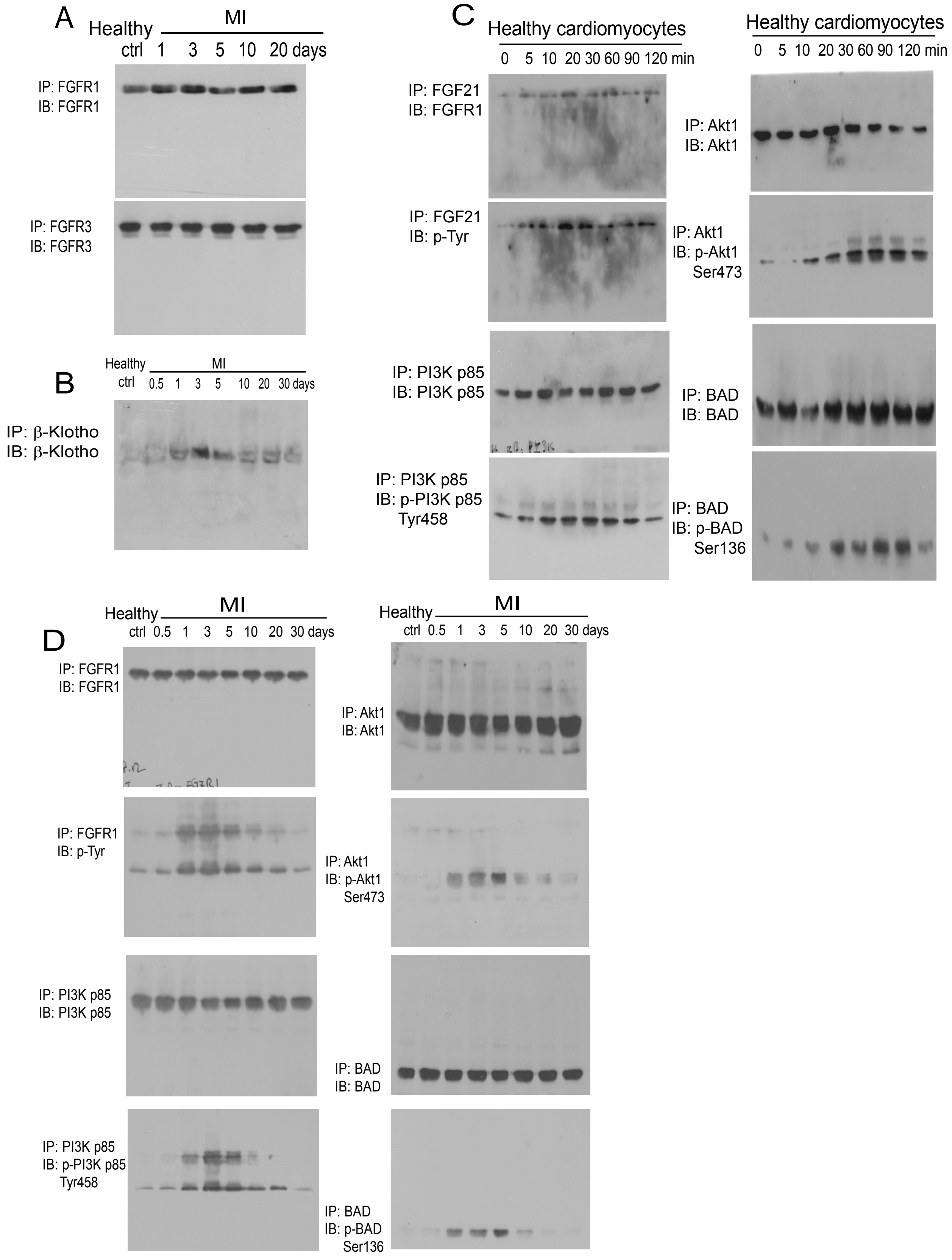

Figure 2. Original immunoblot images for Figure 2 in the main text.

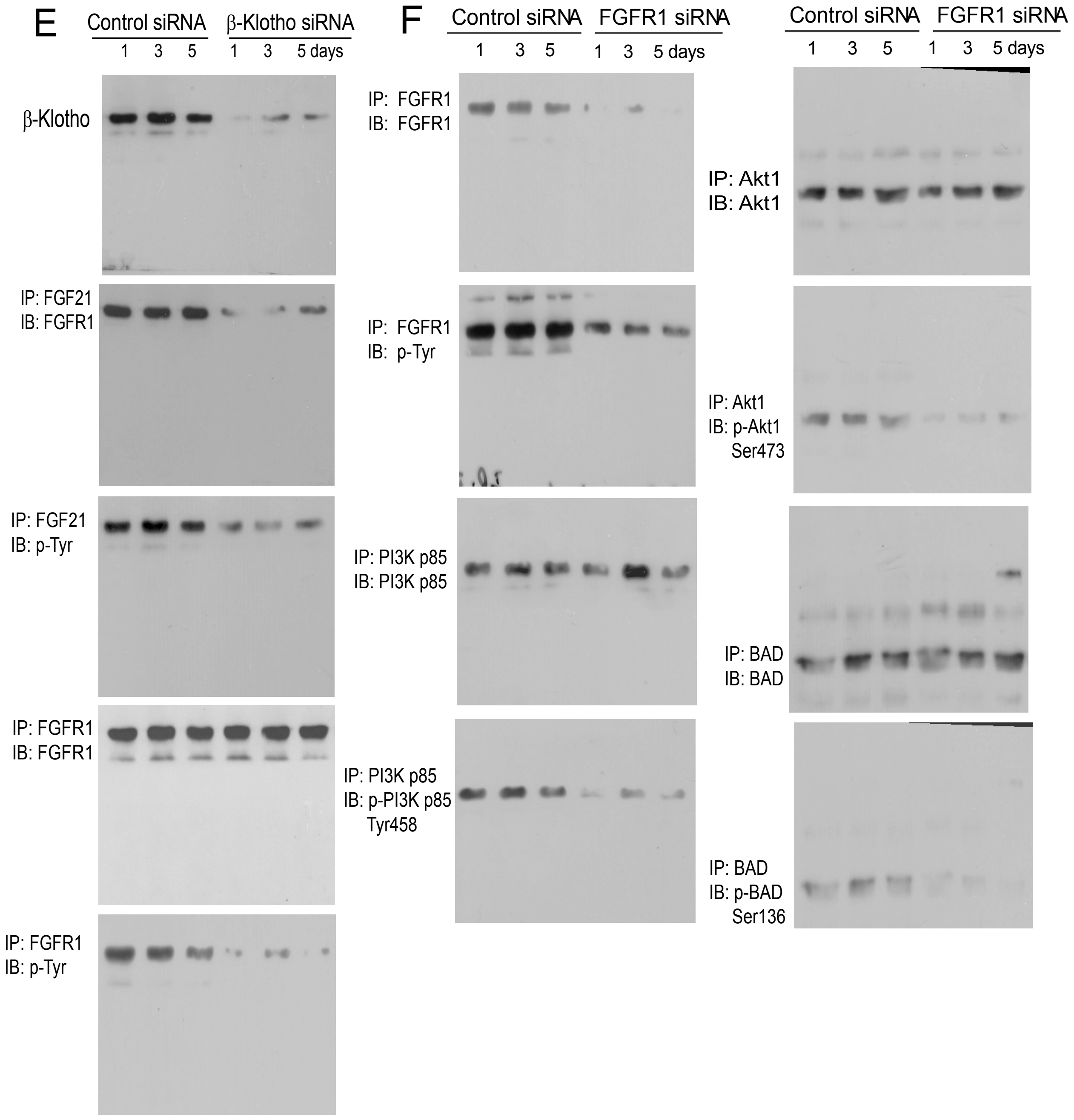

Figure 3. Original immunoblot images for Figure 3 in the main text.

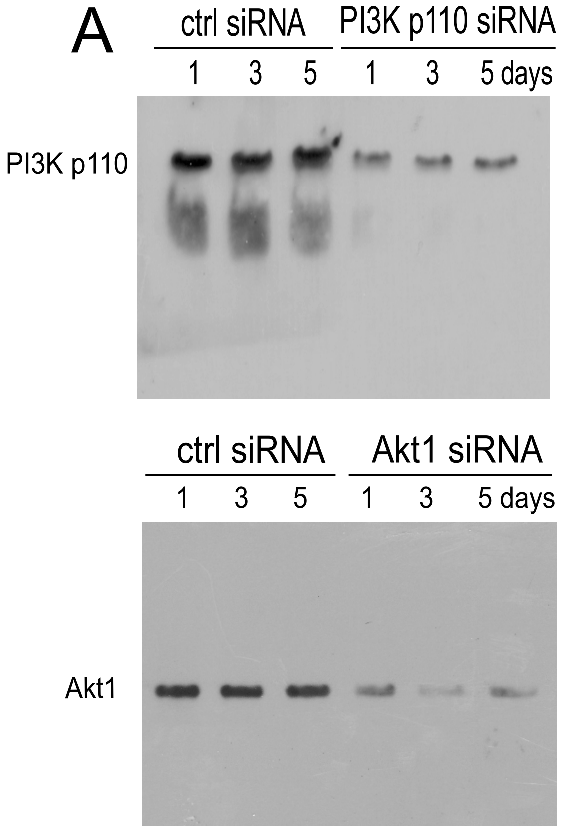

Figure 4. Original immunoblot images for Figure 7 in the main text.

Supplementary Tables

The following tables present P values from post-hoc pairwise multiple comparisons tests by using the Tukey, Bonferroni, Fisher's least-significant-difference, and Scheffé methods for the listed parameters. The most conservative method was selected and used for difference evaluation.

Table 1. Caspase 3 activity in ischemic cardiomyocytes at 24 hrs. The P value from the global ANOVA test (one-way) is < 0.0001.

Treatments	P values			
	Tukey	Bonferroni	Fisher's	Scheffé
a vs. b	< 0.0001	< 0.0001	< 0.0001	< 0.0001
a vs. c	< 0.0001	< 0.0001	< 0.0001	< 0.0001
a vs. d	< 0.0001	< 0.0001	< 0.0001	< 0.0001
b vs. c	< 0.005	< 0.005	< 0.002	< 0.01
b vs. d	> 0.5	> 0.5	> 0.5	> 0.5
c vs. d	< 0.002	< 0.002	< 0.0001	< 0.005

The letters a, b, c, and d represent wild-type sham controls, wild-type myocardial injury (MI), FGF21$^{-/-}$ MI with PBS administration, and FGF21$^{-/-}$ MI with FGF21 administration, respectively.

Table 2. Fraction of TUNEL-positive cell nuclei in the ischemic myocardium at 24 hrs. The P value from the global ANOVA test (one-way) is < 0.0001.

Treatments	P values			
	Tukey	Bonferroni	Fisher's	Scheffé
a vs. b	< 0.0001	< 0.0001	< 0.0001	< 0.0001
a vs. c	< 0.0001	< 0.0001	< 0.0001	< 0.0001
a vs. d	< 0.0001	< 0.0001	< 0.0001	< 0.0001
b vs. c	< 0.0001	< 0.0001	< 0.0001	< 0.0001
b vs. d	< 0.002	< 0.005	< 0.0001	< 0.005
c vs. d	< 0.0001	< 0.0001	< 0.0001	< 0.0001

The letters a, b, c, and d represent wild-type sham controls, wild-type myocardial injury (MI), FGF21$^{-/-}$ MI with PBS administration, and FGF21$^{-/-}$ MI with FGF21 administration, respectively.

Table 3. Fraction of acute myocardial infarcts in reference to the area at risk at 24 hrs. The P value from the global ANOVA test (one-way) is < 0.001.

Treatments	P values			
	Tukey	Bonferroni	Fisher's	Scheffé
a vs. b	< 0.02	< 0.02	< 0.005	< 0.02
a vs. c	< 0.5	< 0.5	< 0.2	< 0.5
b vs. c	< 0.0001	< 0.0001	< 0.0001	< 0.0001

The letters a, b, and c represent wild-type myocardial injury (MI), $FGF21^{-/-}$ MI with PBS administration, and $FGF21^{-/-}$ MI with FGF21 administration, respectively.

Table 4. Fraction of myocardial infarcts at 5, 10, and 30 days.

Table 4.1. Multiple comparisons in reference to times. The P value from the global ANOVA test (two-way) is < 0.0001.

Times (days)	P values			
	Tukey	Bonferroni	Fisher's	Scheffé
5 vs. 10	< 0.0001	< 0.0001	< 0.0001	< 0.0001
5 vs. 30	< 0.0001	< 0.0001	< 0.0001	< 0.0001
10 vs. 30	< 0.0001	< 0.0001	< 0.0001	< 0.0001

Table 4.2. Multiple comparisons in reference to treatments. The P value from the global ANOVA test (two-way) is < 0.0001.

Treatments	P values			
	Tukey	Bonferroni	Fisher's	Scheffé
a vs. b	< 0.02	< 0.02	< 0.01	< 0.05
a vs. c	< 0.0001	< 0.0001	< 0.0001	< 0.0001
c vs. d	< 0.02	< 0.02	< 0.01	< 0.02

The letters a, b, and c represent wild-type myocardial injury (MI), $FGF21^{-/-}$ MI with PBS administration, and $FGF21^{-/-}$ MI with FGF21 administration, respectively.

Table 5. Left ventricular (LV) dp/dt and –dp/dt (absolute value) at 1, 5, 10, and 30 days.

Table 5.1. Multiple comparisons in LV dp/dt in reference to times. The P value from the global ANOVA test (two-way) is > 0.5.

Times (days)	P values			
	Tukey	Bonferroni	Fisher's	Scheffé
1 vs. 5	> 0.5	> 0.5	> 0.5	> 0.5
1 vs. 10	> 0.5	> 0.5	> 0.5	> 0.5
1 vs. 30	> 0.5	> 0.5	< 0.5	> 0.5
5 vs. 10	> 0.5	> 0.5	> 0.5	> 0.5
5 vs. 10	> 0.5	> 0.5	< 0.5	> 0.5
10 vs. 30	> 0.5	> 0.5	> 0.5	> 0.5

Table 5.2. Multiple comparisons in LV dp/dt in reference to treatments. The P value from the global ANOVA test (two-way) is < 0.0001.

Treatments	P values			
	Tukey	Bonferroni	Fisher's	Scheffé
a vs. b	< 0.0001	< 0.0001	< 0.0001	< 0.0001
a vs. c	< 0.0001	< 0.0001	< 0.0001	< 0.0001
a vs. d	< 0.0001	< 0.0001	< 0.0001	< 0.0001
b vs. c	< 0.0001	< 0.0001	< 0.0001	< 0.0001
b vs. d	< 0.0001	< 0.0001	< 0.0001	< 0.0001
c vs. d	< 0.0001	< 0.0001	< 0.0001	< 0.0001

The letters a, b, c, and d represent wild-type sham controls, wild-type myocardial injury (MI), $FGF21^{-/-}$ MI with PBS administration, and $FGF21^{-/-}$ MI with FGF21 administration, respectively.

Table 5.3 Multiple comparisons in LV –dp/dt in reference to times. The P value from the global ANOVA test (two-way) is > 0.5.

Times (days)	P values			
	Tukey	Bonferroni	Fisher's	Scheffé
1 vs. 5	> 0.5	> 0.5	> 0.5	> 0.5
1 vs. 10	> 0.5	> 0.5	> 0.5	> 0.5
1 vs. 30	> 0.5	> 0.5	> 0.5	> 0.5
5 vs. 10	> 0.5	> 0.5	> 0.5	> 0.5
5 vs. 30	> 0.5	> 0.5	> 0.5	> 0.5
10 vs. 30	> 0.5	> 0.5	> 0.5	> 0.5

Table 5.4. Multiple comparisons in LV –dp/dt in reference to treatments. The P value from the global ANOVA test (two-way) is < 0.0001.

Treatments	P values			
	Tukey	Bonferroni	Fisher's	Scheffé
a vs. b	< 0. 0001	< 0.0001	< 0.0001	< 0.0001
a vs. c	< 0.0001	< 0.0001	< 0.0001	< 0.0001
a vs. d	< 0.0001	< 0.0001	< 0.0001	< 0.0001
b vs. c	< 0.01	< 0.01	< 0.002	< 0.02
b vs. d	< 0.0001	< 0.0001	< 0.0001	< 0.0001
c vs. d	< 0.0001	< 0.0001	< 0.0001	< 0.0001

The letters a, b, c, and d represent wild-type sham controls, wild-type myocardial injury (MI), $FGF21^{-/-}$ MI with PBS administration, and $FGF21^{-/-}$ MI with FGF21 administration, respectively.

Table 6. Fractional shortening of the left ventricle at 1, 5, 10, and 30 days.

Table 6.1. Multiple comparisons in reference to times. The P value from the global ANOVA test (two-way) is < 0.1.

Times (days)	P values			
	Tukey	Bonferroni	Fisher's	Scheffé
1 vs. 5	> 0.5	> 0.5	< 0.5	> 0.5
1 vs. 10	> 0.5	> 0.5	< 0.5	> 0.5
1 vs. 30	< 0.5	> 0.5	< 0.2	> 0.5
5 vs. 10	< 0.5	< 0.5	< 0.1	< 0.5
5 vs. 30	< 0.2	< 0.2	< 0.05	< 0.2
10 vs. 30	> 0.5	> 0.5	> 0.5	> 0.5

Table 6.2. Multiple comparisons in reference to treatments. The P value from the global ANOVA test (two-way) is < 0.0001.

Treatments	P values			
	Tukey	Bonferroni	Fisher's	Scheffé
a vs. b	< 0. 0001	< 0.0001	< 0.0001	< 0.0001
a vs. c	< 0.0001	< 0.0001	< 0.0001	< 0.0001
a vs. d	< 0.0001	< 0.0001	< 0.0001	< 0.0001
b vs. c	< 0.0001	< 0.0001	< 0.0001	< 0.0001
b vs. d	< 0.0001	< 0.0001	< 0.0001	< 0.0001
c vs. d	< 0.0001	< 0.0001	< 0.0001	< 0.0001

The letters a, b, c, and d represent wild-type sham controls, wild-type myocardial injury (MI), $FGF21^{-/-}$ MI with PBS administration, and $FGF21^{-/-}$ MI with FGF21 administration, respectively.

Table 7. Influence of siRNA-mediated FGFR1, β-Klotho, PI3K p110, or Akt1 gene silencing on the fraction of myocardial infarcts at day 5. The P value from the global ANOVA test (one-way) is < 0.005.

Treatments	P values			
	Tukey	Bonferroni	Fisher's	Scheffé
a vs. b	< 0.01	< 0.01	< 0.002	< 0.05
a vs. c	< 0.1	< 0.2	< 0.02	< 0.2
a vs. d	< 0.002	< 0.002	< 0.0001	< 0.005
a vs. e	< 0.01	< 0.01	< 0.002	< 0.05
b vs. c	> 0.5	> 0.5	< 0.5	> 0.5
b vs. d	> 0.5	> 0.5	> 0.5	> 0.5
b vs. e	> 0.5	> 0.5	> 0.5	> 0.5
c vs. d	< 0.5	> 0.5	< 0.2	> 0.5
c vs. e	> 0.5	> 0.5	< 0.5	> 0.5
d vs. e	> 0.5	> 0.5	> 0.5	> 0.5

The letters a, b, c, d, and e represent control, FGFR1, β-Klotho, PI3K p110, and Akt1 siRNA treatments, respectively.
